# Culturable *Streptomyces* spp. from high-altitude, oligotrophic North Western Himalaya: a comprehensive study on the diversity, bioactivity and insights into the proteome of potential species

**DOI:** 10.1093/femsec/fiae026

**Published:** 2024-03-04

**Authors:** Aasif Majeed Bhat, Aehtesham Hussain, Qazi Parvaiz Hassan, Adil Bhat

**Affiliations:** Plant Molecular Biology and Biotechnology Division, CSIR-Indian Institute of Integrative Medicine (IIIM), Sanatnagar, Srinagar, Jammu & Kashmir-190005, India; Academy of Scientific and Innovative Research (AcSIR), Ghaziabad, Uttar Pradesh-201002, India; NCMR-National Centre for Cell Science (NCCS), Pune-411007, Maharashtra, India; Plant Molecular Biology and Biotechnology Division, CSIR-Indian Institute of Integrative Medicine (IIIM), Sanatnagar, Srinagar, Jammu & Kashmir-190005, India; Academy of Scientific and Innovative Research (AcSIR), Ghaziabad, Uttar Pradesh-201002, India; Department of Pathology and Laboratory Medicine, University of California Los Angeles, Los Angeles, CA 90095, USA

**Keywords:** antimicrobials, bioactive isolates, phylogeny, proteomics, *Streptomyces* diversity

## Abstract

The increasing global concern of antimicrobial resistance and shortage of new antimicrobials necessitates exploring untapped terrestrial environments for new bioactive microbiome diversity. The low-temperature and oligotrophic North Western Himalaya (NWH) region has a vast diversity of *Streptomyces* with potential antimicrobial properties that remain largely unexplored. This study evaluates the diversity of culturable *Streptomyces* from high-altitude NWH and their potential as a source of new antimicrobials through genus-specific isolation and identification. The results demonstrate a distinct phylogenetic clustering of *Streptomyces* from different sampling regions of NWH, site-specific variation in culturable β-diversity and species commonness with varying intersite bioactivity among different sites. Further, the study optimized the media selection for large-scale culture cultivation in antibiotic production processes and demonstrated the antimicrobial efficacy of *Streptomyces* against a range of pathogens through in vitro bioassays using minimum inhibitory concentration determination and antibiofilm activity. Untargeted label-free proteomic profiling also revealed variable expression of stress-response proteins and antibiotic regulators as a competitive survival strategy for selective antagonistic *Streptomyces*. The findings highlight the potential of NWH in augmenting antimicrobial discovery and combating antimicrobial resistance through the isolation and study of novel bioactive *Streptomyces*.

## Teaser

*Streptomyces* species from habitats of North Western Himalaya display distinct phylogenetic pattern distribution, bioactivity and varied stress-response proteins.

## Introduction

Actinobacteria are known to be the potential reserves for different molecules including antibiotics in clinical use. The genus *Streptomyces* has tremendous bioactive capacity to produce new types of antimicrobials (Schatz et al. 1944, Kardos and Demain 2011). The increase in the rediscovery rate of known antimicrobials from conventional environments (“law of diminishing returns”) has already dried up the antibiotic pipeline and stagnated antibiotic natural products (NPs) drug discovery efforts (Baltz 2006). Novel biosynthetic producers with novel antimicrobial chemical scaffolds are needed to combat resistance and the search for new antimicrobials over three decades has met with very limited success (Newman and Cragg 2016). The call for “new drugs for bad bugs” shifted the focus on NPs drug discovery to extreme soil and aquatic environments (Udwary et al. 2007, Subramani and Aalbersberg 2013, Shah et al. 2017, Hassan et al. 2019, Sivalingam et al. 2019). The genus *Streptomyces* remains an attractive and exciting field in antibiotic drug discovery research and produces a remarkable and diverse array of secondary metabolites, including many antibiotics and anticancer drugs, as well as immune suppressants and antiparasitic agents (Braña et al. 2015). Metagenomic analysis revealed the number of *Streptomyces* reads per Megabase (rpM) to be 129.32, 47.49 and 24.65 rpM from soil, freshwater and marine sources, suggesting that among all three habitats most *Streptomyces* are soil associated (Chevrette et al. 2019). North Western Himalaya (NWH) is the potential growing habitat for different genera of the phylum Actinobacteria, and the genus *Streptomyces* is exciting in its production of different pharmacologically active secondary metabolites (Hussain et al. 2017, Rather et al. 2017, Shah et al. 2017, Singh et al. 2019). *Streptomyces puniceus* AS-13 isolated from Sonamarg Mountain of NWH produces dinactin, a bioactive macrotetrolide compound exhibiting strong antimicrobial (Hussain et al. 2018), antituberculosis (Hussain et al. 2019a) and antitumor activity (Hussain et al. 2019b). *Streptomyces scabrisporus* IIIM55 from NWH produces alborixin, a pharmacologically potential bioactive secondary metabolite that displays significant antiproliferative activity against a panel of cancer cell lines (Shah et al. 2016) and also induces autophagy (Wani et al. 2019). The high altitudes of NWH are stressful environments characterized by cold temperatures, limited vegetation and low-nutrient oligotrophic conditions. *Streptomyces* growing in these stressed environments respond both by antimicrobial production for their competitive survival and regulation of different chaperone systems for proper protein folding and integrity (Bhat et al. 2022). Investigation into the bioactive potential and differential protein expression will provide deeper insights into the *Streptomyces* protein response coupled with antibiotic production.

We selected the genus *Streptomyces* because (i) it is an evolutionarily rich source of most United States Food and Drug Administration-approved and clinically used antimicrobials (Hopwood 2007); (ii) the majority of NP antimicrobials from actinobacteria are produced by *Streptomyces* cultured from the soil (Lewin et al. 2016, Newman and Cragg 2016); and (iii) Biosynthetic Gene Clusters (BGCs) diversity analysis of *Streptomyces* reveals that the genus can produce ~100 000 antimicrobials, however only a small percentage of these have been identified and characterized (Watve et al. 2001, Newman and Cragg 2016). The aim of the current study was (1) to isolate and identify the *Streptomyces* of temperature-constrained and oligotrophic sub-surface soils from NWH; (2) to draw a comparative pattern distribution across different sampling locations; (3) to search for the antibiotic production potential of the identified isolates; and (4) to look for the proteomic diversity of selective bioactive isolates with an emphasis on proteins as stress and antibiotic regulators.

## Materials and methods

### Sampling

**Figure 1.**
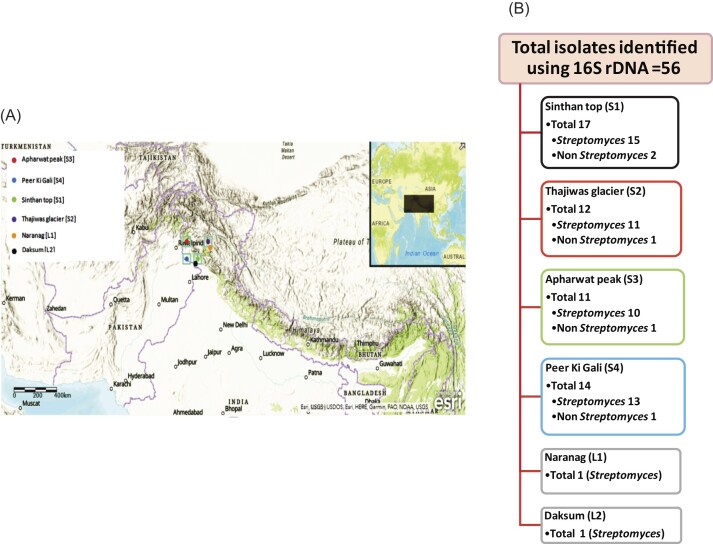
(A) Sampling map drawn using ArcGIS and depicting the latitudinal and longitudinal positioning of different experimental sites. (B) Site-specific details of culturable isolates of majorly *Streptomyces* and a few non-*Streptomyces* from four different sampling sites (S1, S2, S3 and S4). One culture each from Naranag (L1) and Daksun (L2) already isolated by our research group were also taken for 16S-based taxonomic identification.

Four oligotrophic soil sampling sites, S1-S4, from rare (underexplored) and high-altitude NWH were targeted and, using a soil probe, a total of 47 soil samples of different textures were collected from June to September 2016 and 2017 (Table 1 and Fig. 1A). At each sampling site, the surface soil was removed and samples obtained beneath depths of 5–20 cm were sealed in previously labeled sterile polyethylene bags, transported aseptically to the laboratory at an ambient temperature and stored at 4°C. Before processing, all subsamples from different locations of each site were mixed proportionally to form composite samples and labeled accordingly as S1, S2, S3 and S4 to represent four different sampling sites. *Streptomyces* microbes were selectively isolated from these composite samples. Further, we included two *Streptomyces* laboratory cultures already collected and isolated by our research group from Naranag (L1) and Daksum (L2). The details of regional location, DMS coordinates and soil characteristics of sampling sites (S1-S4) and the sites from which laboratory isolates were taken (L1 and L2) are shown in Table 1 and Fig. 1A.

**Table 1.** Numeric detail of four selected sampling sites (S1-S4), their regional location and soil characteristics. Two laboratory *Streptomyces* cultures were isolated each from soil samples collected previously by our research group from Naranag (L1) and Daksum (L2), respectively.

	Isolation sites	No. of samples	Latitude	Longitude	District	Elevation/altitude	pH	Temperature (°C)	Vegetation
Sampling isolates	Sinthan Top (S1)	10	33°34′53.2″N	75°30′37.3″E	Kishtwar	4114 m (13 500 ft)	6.5±0.2	6.5±0.5	Sparse vegetation
	Thajiwas glacier (S2)	12	34°24′20.8″N	76°17′36.1″E	Ganderbal	2800 m (9186 ft)	6.9±0.4	7.5±1	Alpine plants to sparse vegetation
	Apharwat peak (S3)	13	34°02′33.1″N	74°19′30.0″E	Baramulla	4390 m (14 400 ft)	6.3±0.3	8.0±1	Sparse vegetation
	Peer Ki Gali (Pir Panjal Pass) (S4)	12	33°37′48.9″N	74°31′11.3″E	Shopian	3485 m (11 434 ft)	6.8±0.4	6.0±0.4	Alpine meadows to sparse vegetation
Laboratory isolates	Naranag (L1)	NA	34°21′18.1″N	74°58′44.9″E	Ganderbal	2128 m (6982 ft)	6.8±0.4	7.0±0.4	Alpine plants to sparse vegetation
	Daksum (L2)	NA	33°36′36.7″N	75°25′48.5″E	Anantnag	2438 m (8000 ft)	6.5±0.4	7.0±0.4	Sparse vegetation

### Isolation of Actinobacteria

All the composite samples were dried using combined methods of dry heating (120°C, 1 h) (Hayakawa et al. 1991) and drying in a laminar flow hood for 24 h. Actinobacteria were isolated by a standard spread plate method; 1 g of dried soil from each of the composite samples was taken and dilutions of 10^−1^ were prepared using a sterile saline solution. Each of the resultant 10^−1^ dilutions was given a thermal shock at 50°C for 6 min. The preparations were serially diluted down to 10^−6^. Briefly, 200 µl from each dilution was spread over selective isolation media plates of eight types (M1 to M8) in triplicate (Table 2) (Williams et al. 1983). All media were supplemented with the antifungals cycloheximide and nystatin (each at 50 µg/ml) and gram-negative antibiotic nalidixic acid (50 µg/ml). Also, one set of plates was supplemented with novobiocin (25 µg/ml), a gram-positive antibiotic. The inoculated plates were incubated at 28°C for 14 days and examined after every 2 days for the colonies. The putative Actinobacteria colonies taken from selective isolation plates were subcultured and purified at 28°C.

**Table 2.** Composition of different media used for the isolation and growth of Actinobacteria cultures

S. no.	Media (ID)	Composition (g/L)
1	SA (M1)	Streptomyces agar (32 g), sterile water 1 L
2	AIA (M2)	Sodium caseinate (2 g), L-Asparagine (0.1 g), sodium propionate (4 g), dipotassium phosphate (0.5 g), MgSO_4_ (0.1 g), Fe_2_SO_4_ (0.001 g), Agar 15 g, sterile water 1 L, final pH (at 25˚C) 8.1 OR (AIA (21.70 g), 5 ml glycerol in 1 L of water, final pH 8.1
3	ISP2 or Yeast Malt agar (M3)	Yeast extract (4 g), malt extract (10 g), dextrose (4 g), sterile water (1 L), final pH 7.2
4	ISP3 (M4)	ISP3 (38 g), sterile water (1 L)
5	ISP4 (M5)	ISP4 (36.5 g), sterile water (1 L)
6	SC (M6)	Soluble starch (10 g), KNO_3_ (2 g), K_2_HPO_4_ (2 g), NaCl (2 g), tryptone-I (0.3 g), MgSO_4_ (0.05 g), CaCO_3_ (0.02 g), FeSO_4_ (0.01 g), Agar (20 g) sterile water 1 L, final pH 7.3
7	CYPS (M7)	Soluble starch (10 g), casein (10), peptone (1 g), yeast extract (1 g), sterile water 1 L, final pH 7.2
8	KMM (M8)	Dextrose (1 g), KH_2_PO_4_ (0.1 g), NaNO_3_ (0.1 g), KCl (0.1 g), MgSO_4_ (0.1 g), Agar (20 g), sterile water 1 L, final pH 7.2

SA: *Streptomyces* Agar; AIA: Actinomycete Isolation Agar; ISP: International *Streptomyces* Project; SC: Starch Casein; CYPS: Casein, Yeast extract, Peptone and soluble Starch; KMM: Kenknight & Munaier's Medium

### Selection of genus *Streptomyces*

All the isolates were examined for their morphologies, recognized and accordingly selected as putative *Streptomyces* members by their transition from substrate to aerial mycelial morphology patterns, sporulation characteristics, colour pigmentation and also by microscopic observation on Gram staining using binocular Leica microscope model DM 5500. The selective isolates of *Streptomyces* were preserved in 20% glycerol/PBS (v/v) at –80°C.

### DNA isolation

*Streptomyces* cultures were grown in starch casein (SC) broths for 3–4 days and 1.5 ml of cell suspension culture was taken in 2-ml micro centrifuge tubes (or cell mass from bacterial colony was taken and uniformly suspended in 1.5 ml of TE buffer). Cells were harvested by spinning micro-centrifuge tubes for 5 min or until a compact pellet was formed. The pellet was re-suspended in 567-µl TE buffer; 10 µl of 100 mg/ml lysozyme (Thermo Fisher Scientific) was then added and incubated at 37°C for 1–3 h. Next, 50 µl of 10% SDS and 10 µl of 20 mg/ml Proteinase K (Promega) were added before lysis by incubation at 55°C for 1 h. Then 100 µl of 5 M NaCl and 80 µl of CTAB/NaCl were added, the tubes were mixed thoroughly and incubated at 65°C for 20 min. The tubes were cooled to 28°C and an equal volume of chloroform: isoamyl alcohol (24:1 ratio) was added and the tubes were gently shaken until uniformly white and spun at 10 000 r/m for 10 min. After centrifugation, the top aqueous layer was transferred to a new micro-centrifuge tube and extracted again with an equal volume of phenol: chloroform: isoamyl alcohol (25:24:1 ratio). The top layer was again transferred to another tube and precipitated with 0.6 volume of isopropanol. The precipitate was centrifuged at 10,000 rpm for 10–20 min and the pellet was washed and spun twice in 500 µl of 70% ethanol at 10 000 r/m for 5 min and resuspended in 100-µl TE buffer. DNA was quantified (NanoDrop Spectrophotometer ND-1000, Thermo Scientific), run on 0.8% gel to check for purity and verify high molecular weight.

### Identification by 16S rDNA sequencing and phylogenetic affiliations

After genomic DNA extraction and quantification, nearly complete 16S rRNA gene was amplified using a set of universal primers: 27F (5′-AGAGTTTGATCCTGGCTCAG-3′) and 1492R (5′-GGTTACCTTGTTACGACT T-3′) of hyper-variable regions V1-V9. PCR was performed in a 96-well Thermal cycler (Applied Biosystems) in a standard 25-µL reaction with 1-µL DNA template (<50 ng µL^−1^), 2.5-µL Taq polymerase assay buffer, 1 µL each of the 10-µM primers, 2 µL of dNTPs, 1.5 µL of MgCl, 0.25 µL of Taq DNA Polymerase (QIAGEN) and 15.75 µL of nuclease free water. Amplification profile with initial denaturation at 94°C (3 min) was followed by 30 cycles of denaturation at 94°C (30 s), annealing at 55°C (30 s), extension at 72°C (90 s) and a final extension at 72°C (5 min), followed by infinite hold at 10°C. Amplicons were confirmed on 1% agarose electrophoresis gel prior to cleanup using the in-house PEG/NaCl purification system. The 16S rRNA gene was sequenced by ABI 3730xl Genetic Analyzer (Applied Biosystems) using the sequencing primers 27F, 536F, 946F, 907R, 518R and 1492R. The sequence chromatograms of 16S rRNA gene of each strain were checked and assembled by SeqMan Pro assembler. The resultant sequences (∼1350 nt) were blasted with EzBioCloud Database and the top 50 or maximum number of hits (whichever was possible) were taken. 16S rRNA gene query sequence alignment with blast hits was performed using MEGA 11-based MUSCLE Alignment explorer. The evolutionary history was deduced using the Neighbor-Joining method and the percentage bootstrap consensus tree using 1000 replicates was generated to represent the evolutionary history of the analyzed taxa (Felsenstein 1985, Saitou and Nei 1987). The Maximum Composite Likelihood method was used to compute evolutionary distances and the final evolutionary analysis was conducted in MEGA 11 v. 11.0.11 (Tamura et al. 2004, Tamura et al. 2021). The tree was visualized, manipulated and annotated in The Interactive Tree Of Life (iTOL v. 6.6) (Letunic and Bork 2021).

### Media optimization of highly bioactive strains

Following the preliminary screening by the Agar Well Diffusion method against all the isolates, the 12 highly bioactive strains were selected and inoculated in triplicate in four different production media: SC (Starch Casein), KMM (Kenknight & Munaier’s Medium), ISP2 (International Streptomyces Project 2) and CYPS (Casein, Yeast extract, Peptone and soluble Starch) (Table 2) (each containing 250 ml of medium in 1000-ml Erlenmeyer flasks) and incubated at 28°C for 14 days at 180 r/m. The fermented broths were centrifuged (Eppenforf Centrifuge 5810 R) at 4000 r/m for 12 min, followed by filtration. The filtrates were mixed with ethyl acetate solvent (2X), vigorously shaken and extracted exhaustively. The supernatant organic layers were concentrated on Rotavapor (R-215, BUCHI). The concentrated extracts were dried and quantified.

### Preparation of extracts of highly bioactive strains in optimized media

The *Streptomyces* isolates were grown as culture broths (300 ml in 1000-ml Erlenmeyer flasks) in different optimized media selected against each strain and incubated at 28°C for 8–10 days in an orbital shaker (Forma) with 180 r/m. The fermented broths were centrifuged at 7000 r/m for 8 min to pellet down the mycelial cell mass. The cell-free supernatants were solvent extracted by ethyl acetate (2X). After exhaustive extraction, the organic layers were concentrated on a rotary evaporator (Rotavapor, R‐215; BUCHI, Switzerland). The extracted concentrates were quantified and evaluated for antimicrobial activity.

### Test microorganisms

The panel of test microorganisms, *Escherichia coli* (ATCC 25922), *Pseudomonas aeruginosa* (ATCC 27853), *Klebsilla pneumonia* (ATCC 700603), *Staphylococcus aureus* (ATCC 25923), *Enterococcus faecalis* (ATCC 29212) and *Micrococcus luteus* (ATCC 10240), used in the study were acquired from American Type Culture Collection (ATCC), USA.

### Minimum inhibitory concentration measurements

The minimum inhibitory concentrations (MICs) of extracts against test strains of microorganisms were determined by the broth microdilution method (Wiegand et al. 2008), prescribed under the guidelines of Clinical and Laboratory Standards Institute (CLSI) procedures, M07, 11th edition (2018–9) (Humphries et al. 2018). Briefly, the stock solutions (50 mg ml^−1^) of extracts were prepared in DMSO and the reference drug, ciprofloxacin (Sigma-Aldrich), was dissolved in 0.1 N HCl at 25 mg/ml. Aliquots of both the extracts and reference drug were further diluted with Muller Hinton broth (MHB) to form working concentrations. Starting with the first well, 2-fold serial dilution in 200-µl volumes and bacterial inoculum (50 µl) was added into each well of a 96-well plate with the final cell density of 1 × 10^5^ CFUs/ml. Media containing microbial cells only and ciprofloxacin served as negative and positive controls, respectively. The plates were incubated at 37°C for 24 h and visualized to determine the MICs. The MIC was defined as the lowest concentration that prevented the visible microbial growth. Each MIC (in µg/ml) test was performed twice in triplicate.

### Biofilm assay

Micro titre plate biofilm assay was used to visualize the attachment pattern of microbial cells to an abiotic surface. Briefly, cultures of different pathogenic microorganisms were grown overnight in MHB then diluted to 1:100 in fresh Mueller Hinton medium and 50 µL of diluted culture was added into each well in 96-well plates. DMSO-dissolved ethyl acetate extracts of bioactive preparations were added into each well to make the final concentration up to 100 µg ml^−1^, except for culture control (negative control) and media control wells. Ciprofloxacin served as the positive control. The plates were incubated at 37°C for 48 h. Then the plates were briskly shaken in an overturned position to remove planktonic bacteria from each well then submerged three times in three separate wash trays containing 2–3 in of tap water. The plates were then vigorously shaken to remove any liquid. Next, 125 µL of 0.1% crystal violet solution was added to each well and stained for 10 min at room temperature (RT). The plates were then again vigorously shaken in wash trays to remove excess stain, inverted and tapped on paper towels to remove excess liquid and allowed to air dry. After drying, 250 µL of 30% acetic acid solvent was added to each well to solubilize the dye by incubating at 10–15 min at RT. The contents in each well were briefly mixed by pipetting and 125 µL of dye-solvent solution from each well was transferred to separate flat bottom 96-well plates. The optical density (OD) of each well was measured at 600 nm. The measurements for each well were replicated thrice and the mean absorbance values were calculated. Biofilm inhibition was calculated using the formula:


\begin{eqnarray*}
{\rm Percentage\,\, inhibition} &=& \frac{{\rm OD}_{\rm Negative\,\,control} - {\rm OD}_{\rm Experimental}}{{\rm OD}_{\rm Negative\,\,control}}\,\, \times \,100
\end{eqnarray*}

### Proteomics and systems biology

Next, 100 µL of each selected *Streptomyces* culture grown to stationary phase in optimized media at 28°C for 6–8 days was taken and 4% of SDS was added and kept for 15 min at 95°C. Total proteins in each sample were then precipitated by adding ice cold 6X acetone and centrifuged for 10 min at 13 000 r/m. The proteins precipitated were then rediscovered in ammonium bicarbonate buffer of pH 7 and subjected to Liquid Chromatography with tandem Mass Spectrometry (LC-MS/MS) proteomic evaluation. Then 100-µL equivalent of protein from each sample was subjected to reduction and alkylation followed by trypsin digestion at 37°C for 16–20 h using a sequencing grade modified trypsin: proteins ratio of 1 µg: 20 µg w/w. The cleaved peptides were cleaned by desalting and subjected to downstream LC-MS/MS analysis using the following parameters: peptide elution from 3 to 95% gradient at a flow rate of 300 µL/ml with buffer B (aqueous 80% acetonitrile in 0.1% formic acid). The elution gradient was carried for ~60 min on a 25-cm analytical C18 column (C18, 3 mm, 100 A˚). The peptide ionization was performed by nano-electrospray followed by tandem MS/MS on a Q-ExactiveTM Plus (Thermo Fisher Scientific, San Jose, CA, USA). The ionized peptides were then analyzed using collision-induced dissociation mode in a mass spectrometer with the electrospray voltage of 2.3 kV. Using orbitrap the complete scan analysis of MS spectra was performed with a resolution of 70 000 from *m/z* 350 to 1800 and further analysis on MS/MS data was performed in Proteome Discoverer (version 2.0, Thermo Fisher Scientific, Waltham, MA, USA) using the UniProt database. Significant proteins at a threshold *P* < 0.05 were identified and subjected to statistical analysis using MetaboAnalyst version 5.0.

## Results

### Culturable *Streptomyces* from NWH soils display overall phylogenetic clustering, site-specific phylogenetic clustering, culturable β-diversity variation and species commonness among different sampling locations

Two hundred and fifty-four representative isolates putatively assigned to the phylum Actinobacteria were purified from the isolation plates. Based on the morphological and microscopic examination, 56 culturable isolates showing striking structural similarities to *Streptomyces* morphology were isolated from different sampling sites: 54 from four different sampling sites: 17 from Sinthan top (S1), 12 from Thajiwas glacier (S2), 11 from Apharwat peak (S3) and 14 from Peer Ki Gali (S4); and two laboratory isolates, ASQP 76 and ASQP 48, were already isolated by our research group from Naranag (L1) and Daksun (L2), respectively (Fig. 1A and B). The reason for incorporation and inclusion of these two isolates from L1 and L2 was due to the morphologically similarity characteristics found between these two laboratory and two sampling isolates, which may give an important idea about the widespread presence of similar *Streptomyces* species. The media-specific isolation of cultures on agar plates yielded three isolates on Streptomyces agar (M1), four on Actinomycete Isolation Agar (M2), 11 on ISP2 (M3), two on ISP3 (M4), three on ISP4 (M5), 11 on SCA (Starch Casein Agar) (M6), nine on CYPS (M7) and 13 on KMM (M8). Thus, most of the isolates from these high-altitude regions were cultured on media using KMM, SCA, ISP2 and CYPS. All the isolated strains were further identified by 16S rRNA gene-based identification. Following molecular identification, all isolates except five (ASQP 51 and ASQP 128: *Microbacterium* spp.; ASQP a3 and ASQP a5: *Nocardiopsis* spp.; and ASQP 74: *Kitasatospora* sp.) were taxonomically found to belong to the genus *Streptomyces* (56–5=51 culturable *Streptomyces* isolates) (Supplementary Table 1). The five non-*Streptomyces* isolates showed a physical and microscopic appearance that was similar to *Streptomyces*, but upon molecular identification were found to be members of three different genera. Thus, the average hit rate for rediscovery of *Streptomyces* from these four sampling sites was 51/254=20%. Becasue we focused our studies only on *Streptomyces*, the following were the significant observations made from the phylogenetic affiliations of identified isolates.

#### 16S rRNA lacks Streptomyces taxonomic resolution below genus level

Taxonomic classification of each taxon was deduced based on the results of three analytical approaches: (1) maximum percentage similarity of query sequence to top blast hit/s; (2) individual phylogenetic affiliations of query sequence; and (3) concatenation of all the query sequences with their hits and drawing the combined phylogeny. The final conclusion about the proper taxonomic identification of each taxon was reached after analyzing the results of all three approaches (Supplementary Fig. S1, Supplementary Table 2 and Supplementary Table 3). Of the 51 *Streptomyces* isolated from different sampling locations, only 23 were phylogenetically identified to species level; the remaining 28 taxa were classified up to genus level only [e.g. *Streptomyces* sp. ASQP 4 (1425 bp) showed 100% similarity with five *Streptomyces* species, namely, *S. badius, S. globisporous, S. sindenensis, S. parvus* and *S. pluricolorescens; Streptomyces* sp. ASQP 15 (1344 bp) showed 99.03% similarity with three *Streptomyces* species, namely, *S. badius, S. parvus* and *S. sindenensis*; and *Streptomyces* sp. ASQP 52 (1394 bp) showed 100% similarity with two *Streptomyces* species, namely, *S. microflavus* and *S. fulvorobeus*]. Supplementary Fig. S2 and Supplementary Table 2 show the similarity percentages along with their amplified base pairs sequences. Thus, most of these species showed a percentage similarity with their top hits above 99%; however, they could not be taxonomically assigned to species level, highlighting that 16S rRNA alone is insufficient for proper *Streptomyces* identification and majorly cannot resolve species level delineation in such bacteria.

NWH has the potential to produce novel (bioactive) *Streptomyces. Streptomyces* sp. ASQP 6 (98.64% identity to *Streptomyces pseudovenezuelae*), ASQP 94 (98.96% identity to *Streptomyces fagopyri*) and a bioactive strain, ASQP 123a (98.71% identity to *Streptomyces pseudovenezuelae*), displayed sequence homology similarity well below the cut-off limit of 99%, suggestive of potential for isolation of novel *Streptomyces* taxa from NWH. Therefore, the taxonomic status of *Streptomyces* spp. strains whose taxonomic identification could not be resolved to species level and in which each query taxon showed a percentage sequence similarity to two or more close species, thus warrant further taxonomic resolution to delineate some of these *Streptomyces* as a new species, suggestive of the importance of NWH as a region of potentially novel *Streptomyces* producers (Supplementary Fig. S2).

#### Phylogenetic clustering of Streptomyces of NWH

**Figure 2.**
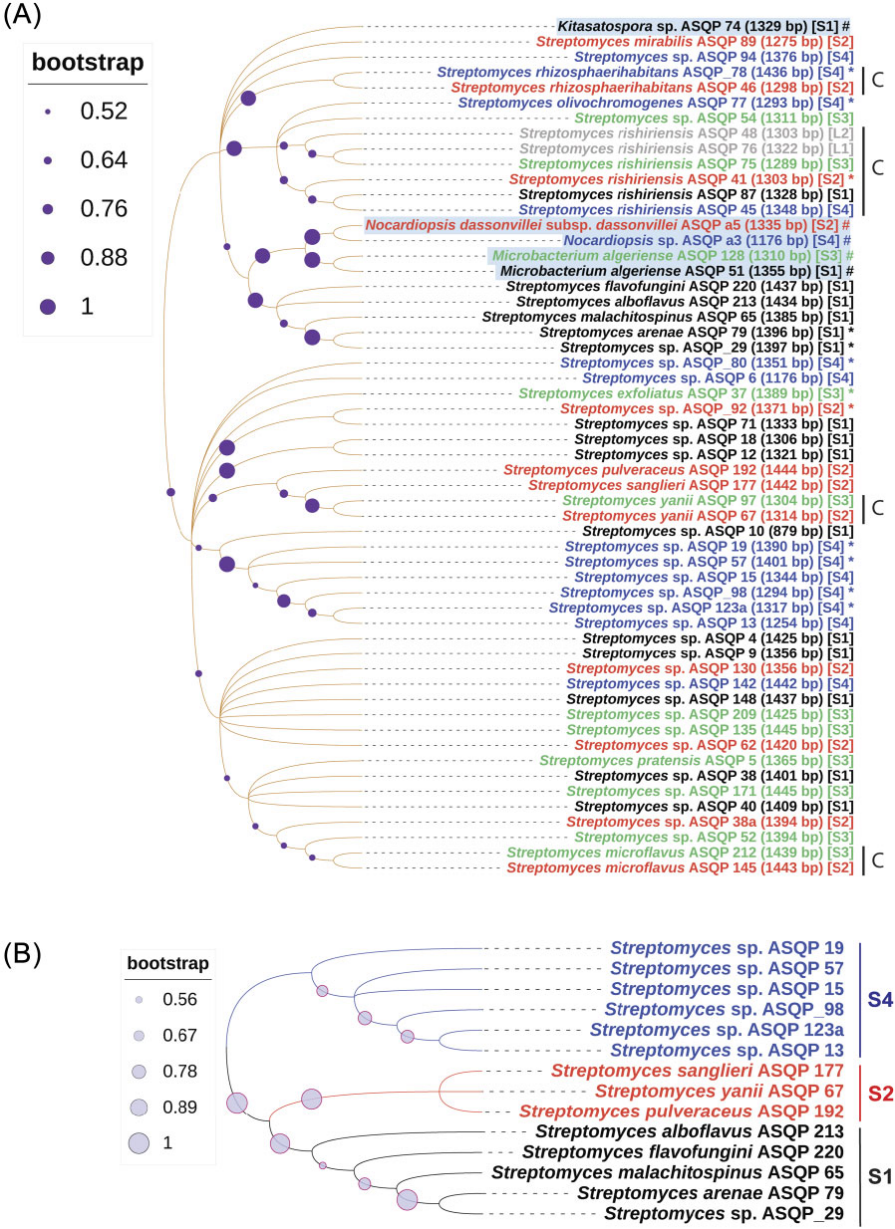
(A) Phylogenetic affiliations using 16S rDNA nucleotide sequences of culturable 56 isolates. The evolutionary history was deduced using the Neighbor-Joining method and the percentage bootstrap consensus tree using 1000 replicates was generated to represent the evolutionary history of the analyzed taxa. The Maximum Composite Likelihood method was used to compute evolutionary distances and the final evolutionary analysis was conducted in MEGA 11. The tree was visualized, manipulated and annotated in The Interactive Tree Of Life (iTOL). Each taxon is shown with organism nomenclature, strain ID, number of base pairs amplified and the sampling site from which the taxon is isolated. Bioactive isolates are marked with an asterisk (*) and non-*Streptomyces* isolates are shown with a gray background color and are marked with #. Different font colors represent taxa isolated from different sampling sites: black for S1 (Sinthan top), red for S2 (Thajiwas glacier), green from S3 (Apharwat peak), blue from S4 (Peer Ki Gali) and gray for both L1 (Naranag) and L2 (Daksun). The capital “C” letter represents common species from different sampling regions. (B) Site-specific phylogenetic clustering among different sampling locations.

*Streptomyces* of NWH are grouped together into many separate phylogenetic clades, suggesting monophyletic origin and taxonomic species similarity. Supplementary Fig. S1 shows several of such monophyletic clades (e.g. isolates with the strain IDs ASQP 4, ASQP 62, ASQP 130, ASQP 38, ASQP 40, ASQP 9 and ASQP 171 are clustered together in separate clades). Likewise, we noticed substantial phylogenetic clustering in clades bearing the strain IDs ASQP 76, ASQP 75, ASQP 45, ASQP 87 and ASQP 41, as well as clades bearing the strain IDs ASQP 10, ASQP 15, ASQP 19, ASQP_98, ASQP 57, ASQP 123a and ASQP 13. This phylogenetic taxonomic clustering is also supported by inter-comparative taxonomic study of isolated strains (Fig. 2A).

#### Site-specific phylogenetic clustering

Moderate levels of site-specific phylogenetic clustering were observed among different sampling locations and *Streptomyces* isolated from different sites tend to cluster in different clades (e.g. *Streptomyces* spp. with the strain IDs ASQP 13, ASQP 123a, ASQP_98, ASQP 15, ASQP 57 and ASQP 19 clustered in a single phylogenetic clade and were all isolated from the S4 site). Likewise, site-specific phylogenetic clustering was observed in ASQP_29, ASQP 79, ASQP 65, ASQP 213 and ASQP 220, which were all isolated from the S1 site. With the one exception of ASQP 97 isolated from S3, phylogenetic clustering was also observed in ASQP 192, ASQP 177 and ASQP 67 isolated from S2 (Fig. 2B).

#### Culturable β-diversity variation among different sampling locations

With some exceptions of those *Streptomyces* common to two or more sampling locations (see the section below), *Streptomyces* culturable beta diversity (β-diversity) varies moderately with respect to biogeographically distant places. Of the 15 *Streptomyces* isolated from S1, four taxa with the strain IDs ASQP 65, ASQP 79, ASQP 213 and ASQP 220 were only found from S1 and not from any other sampling site. Likewise, of the 11, 10 and 13 *Streptomyces* isolated from S2, S3 and S4, respectively, three taxa with the strain IDs ASQP 89, ASQP 177 and ASQP 192, two taxa with the strain IDs ASQP 5 and ASQP 37, and one taxon with the strain ID ASQP 77, were only found from S2, S3 and S4, respectively (Supplementary Table 3 and Fig. 2A).

#### Species commonness among different sampling locations

Interspecies comparison revealed that common *Streptomyces* species (with varying bioactive potential, marked with an asterisk) were isolated from different bio geographic sampling locations. *Streptomyces rishiriensis* was isolated from all four sampling locations (*S. rishiriensis*; ASQP 87-S1, ASQP 41*-S2, ASQP 75-S3, ASQP 45-S4). *Streptomyces rhizosphaerihabitans* was isolated from both S2 and S4 (ASQP 46-S2, ASQP 78*-S4). *Streptomyces microflavus* (ASQP 145 and ASQP 212) and *S. yanii* (ASQP 67 and ASQP 97) were both isolated from S2 and S3, respectively (the capital “C” letter against common species among different sites in Fig. 2A). Also, two laboratory isolates L1 and L2 upon molecular identification were found to be *S. rhishiriensis*. The characteristic feature of species commonness is that same species isolated from different sites can show varying bioactive potential. Although *S. rishiriensis* was isolated from all the sampling sites, only one strain (ASQP 41) collected from the Thajiwas glacier (S2) showed antimicrobial inhibitory bioactivity. Likewise, *S. rhizosphaerihabitans* was isolated from two sampling sites, S2 and S4, but only the one isolated from Peer Ki Gali (S4) displayed antimicrobial potential (Fig. 2A).

The above analysis of phylogenetic clustering pattern, β-diversity and intraspecies commonness numbers may change, however, because of changes in species delineation and proper identification of isolates not assigned with species-level identification.

### Bioactive *Streptomyces* displayed optimized growth in different media for metabolite production

**Figure 3.**
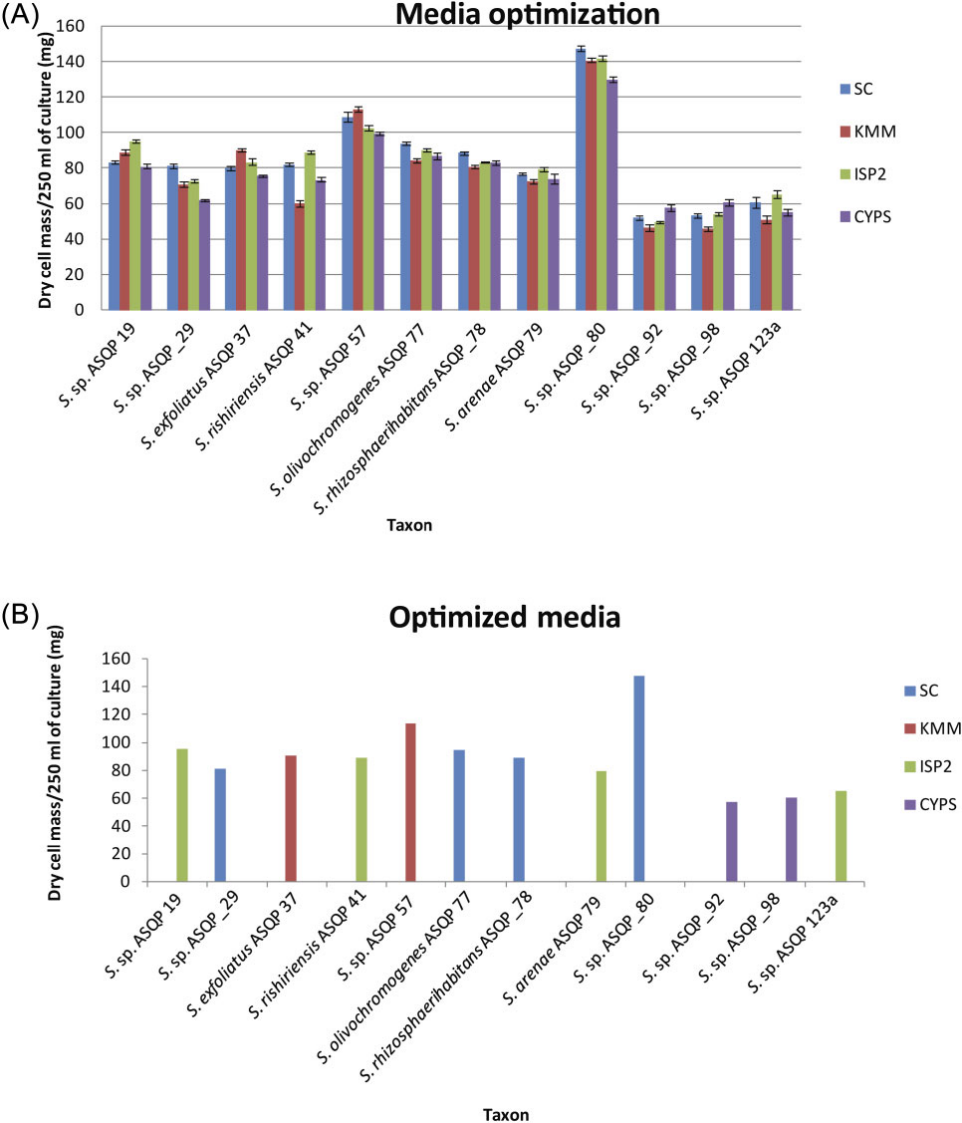
(A) Growth optima calculated as dry cell mass in 250 ml of broth culture of 12 bioactive *Streptomyces* grown in four different media: starch casein medium (SC), Kenknight & Munaier's Medium (KMM), Yeast Malt medium (ISP2) and Casein Yeast Peptone Starch medium (CYPS). The error bars represent the standard error of mean (SEM). (B) Optimized growth of *Streptomyces* isolates against a particular medium.

The quantification of concentrated ethyl acetate extracts of 12 bioactive isolates showed different bacterial growths in four production media, as shown by the dry cell mass (Fig. 3A). Among the four selected media, isolates with the strain IDs ASQP 19, ASQP 41, ASQP 79 and ASQP 123a showed optimum growth in ISP2; ASQP_29, ASQP 77, ASQP_78 and ASQP_80 in SC; ASQP 37 and ASQP 57 in KMM and the growth optimum for ASQP_92 and ASQP_98 was shown in CYPS (Fig. 3B). Dry cell mass production was highest in ASQP_80 and lowest in ASQP_92. Although the dry cell mass content for each isolate cultured in 250 ml of all four production media seems not so different, this difference in the fermentation perspective can be huge where hundreds and thousands of liters of production media are employed for large-scale industrial culture cultivation and bioactive antimicrobial secretion.

### Bioactive *Streptomyces* from NWH possess promising antibacterial and antibiofilm potential

#### In vitro antimicrobial activity of potential Streptomyces

**Figure 4.**
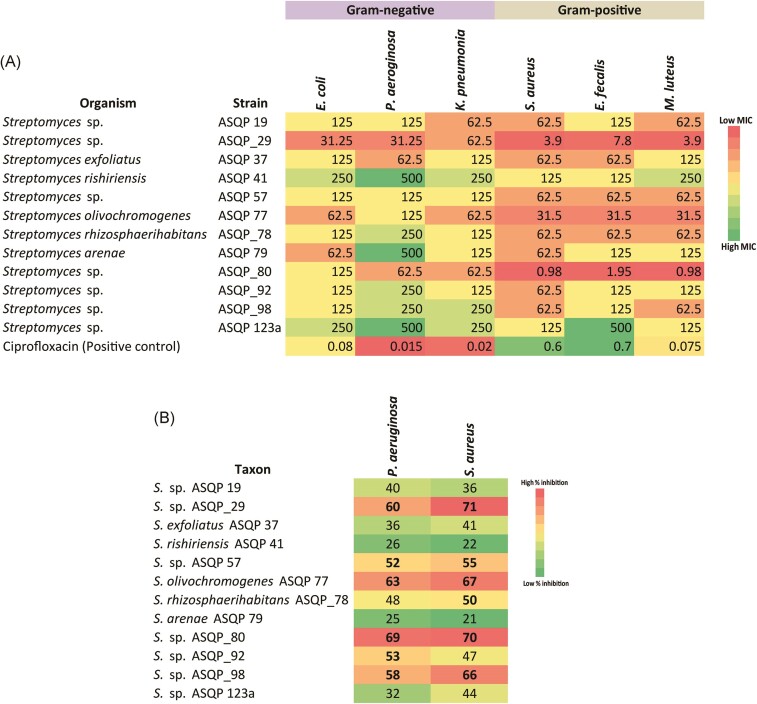
Antimicrobial inhibition and antibiofilm activity of antagonistic *Streptomyces* against a panel of pathogens. (A) In vitro antimicrobial potential measured as minimum inhibitory concentration (MIC) in µg/ml of 12 bioactive isolates against three gram-positive and three gram-negative test pathogens. Color gradient from green to red denotes lower to higher antimicrobial inhibition. Ciprofloxacin taken as a positive control displayed MIC values against different pathogens as *Escherichia coli*: 0.08 µg ml^−1^, *Pseudomonas aeruginosa*: 0.015 µg ml^−1^, *Klebsiella pneumonia*: 0.02 µg ml^−1^, *Staphylococcus aureus*: 0.6 µg ml^−1^, *Enterococcus faecalis*: 0.7 µg ml^−1^ and *Micrococcus luteus*: 0.075 µg ml^−1^. (B) In vitro biofilm inhibition (as percentage units) by antagonistic *Streptomyces* isolates against a gram-positive (*Staphylococcus aureus*) and a gram-negative (*Pseudomonas aeruginosa*) pathogen. Color gradient from green to red denotes lower to higher percentage biofilm inhibition. As per established criterion, percentage values ≥50% suggest good biofilm inhibition.

Next, we evaluated the antimicrobial potential of all *Streptomyces* spp. using well diffusion assays and then determined MICs (in µg/ml) of 12 bioactive *Streptomyces* species with 10 repetitions of 2-fold serial dilution concentration ranges set between 0.49 and 500 µg ml^−1^. All isolates showed broad spectrum antimicrobial activity against both gram-positive and gram-negative pathogens, with MIC values ranging from 500 µg ml^−1^ to as low as 0.98 µg ml^−1^ (Fig. 4A). Two *Streptomyces* strains, *S*. sp. ASQP_29 and *S*. sp. ASQP_80, displayed strong antimicrobial activity and were maximally antagonistic against the gram-positive pathogens *Staphylococcus aureus* and *Micrococcus luteus*. The lowest MICs reported in these two strains were 3.9 µg ml^−1^ for *S*. sp. ASQP_29 and 0.98 µg ml^−1^ for *S*. sp. ASQP_80. The MIC values of the rest of the isolates were comparatively higher than the above two strains; however, all the bioactive isolates were comparatively more antagonistic against gram-positive pathogens than gram-negative pathogens.

#### Antibiofilm activity of bioactive Streptomyces

We subsequently performed antibiofilm assay and evaluated the ability of 12 bioactive *Streptomyces* isolates to prevent or inhibit biofilm formation against a representative gram-positive (*Staphylococcus aureus*) and a gram-negative (*Pseudomonas aeruginosa*) pathogen. The 12 bioactive isolates varied in respect of the prevention of biofilm formation, however all displayed positive results suggest biofilm inhibition (Fig. 4B). The top six isolates strongly antagonistic against the biofilm formation by *Pseudomonas aeruginosa* were ASQP_29, ASQP 57, ASQP 77, ASQP_80, ASQP_92 and ASQP_98, and also the six isolates that displayed strong activity against the biofilm formation by *Staphylococcus aureus* were ASQP_29, ASQP 57, ASQP 77, ASQP_78, ASQP_80 and ASQP_98 with percentage inhibition values ≥50%; however, the antibiofilm activity of the remaining six extracts against both pathogens was comparatively <50%.

#### Site-specific phylogenetic clustering of bioactive isolates

**Figure 5.**
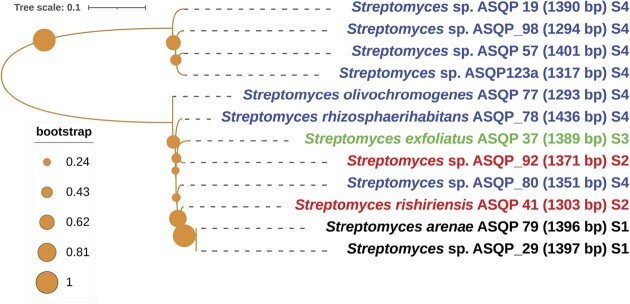
Phylogenetic affiliations using 16S rDNA nucleotide sequences of 12 bioactive culturable *Streptomyces*. The evolutionary history was deduced using the Neighbor-Joining method and the percentage bootstrap consensus tree using 1000 replicates was generated to represent the evolutionary history of the analyzed taxa. The Maximum Composite Likelihood method was used to compute evolutionary distances and the final evolutionary analysis was conducted in MEGA 11. The tree was visualized, manipulated and annotated in The Interactive Tree Of Life (iTOL). Each taxon is shown with organism nomenclature, strain ID, number of base pairs amplified and the sampling site from which the taxon is isolated. Different font colors represent taxa isolated from different sampling sites: black for S1 (Sinthan top), red for S2 (Thajiwas glacier), green from S3 (Apharwat peak) and blue from S4 (Peer Ki Gali).

Site-specific phylogenetic clustering was also noticed by evaluating the phylogenetic affiliations of only 12 bioactive *Streptomyces* species. Of the seven bioactive *Streptomyces* collected from Peer Ki Gali (S4), four isolates, ASQP 19, ASQP_98, ASQP 57 and ASQP 123a, are clustered into a single clade. Further, both the bioactive isolates ASQP_29 and ASQP 79 from Sinthan top (S1) are also grouped into a single separate clade (Fig. 5).

### Proteomic phenotype of bioactive *Streptomyces* is predictive of both clustering and strain-specific variability in expression of some antibiotic response regulators and stress-responsive proteins

**Figure 6.**
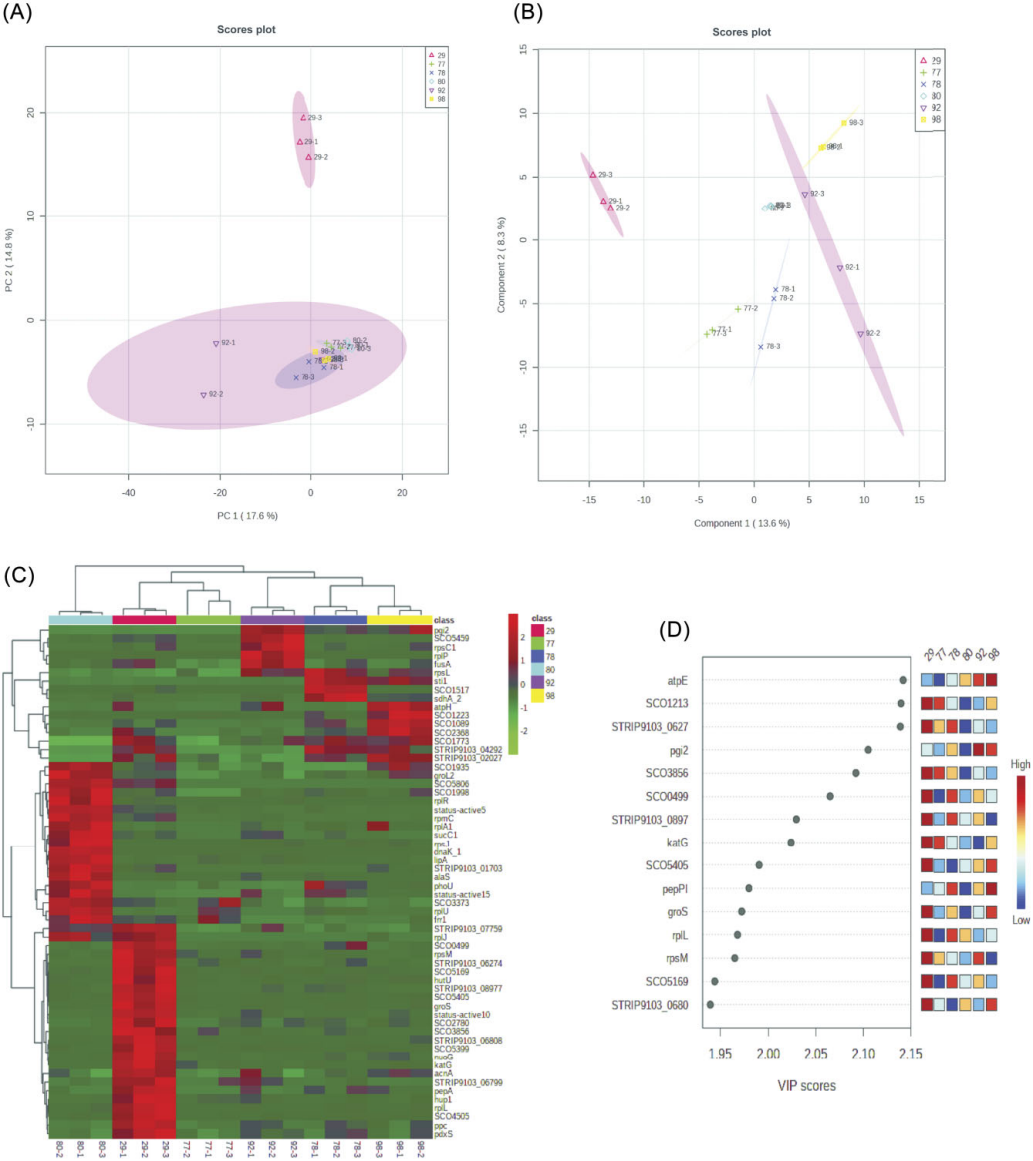
Proteomic phenotype of six bioactive *Streptomyces* (ASQP_29, ASQP_77, ASQP_78, ASQP_80, ASQP_92 and ASQP_98). (A) Scores plot for 2D Principal Component Analysis displaying segregation and overlapping variability among six bioactive *Streptomyces* spp. The plot shows 32.4% of total variance. Variance proportion for PC1 along the x-axis is 17.6% and for PC2 along the y-axis is 14.8%. (B) 2D scores plot for partial least square discriminant analysis (PLS-DA) using MetaboAnalyst 5.0, displaying clear segregation of six bioactive *Streptomyces*. A five-component model was performed by PLS-DA. The plot shows six different samples in different colors. Ovals display 95% confidence intervals for all six samples. The diagram shows a scatterplot for two components of the greatest variation. Similar observations will fall close to each other and display a cluster-like pattern. Component 1 (x-axis) contains 13.8 of total variation and Component 2 contains 8.3%. (C) Hierarchical clustering (heatmap) analysis was performed for six different *Streptomyces*, comparing 60 Benjamini–Hochberg FDR correction t-test *P* value (<0.05) passing proteins. For clustering, MetaboAnalyst 5.0 was used. The horizontal axis represents all the samples analyzed in the study and the vertical axis is UniProt accessions for 60 proteins. On top of the heatmap are six different *Streptomyces* samples, each represented by a different color. A dendrogram for different *Streptomyces*, each in triplicate, is shown on top of the heatmap and the protein dendrogram is on the left-hand side of the heatmap. Color gradient from dark blue to dark red denotes lower to higher expression. (D) Variable Importance in Projection (VIP) plot displays the top 15 most important proteins identified by PLS-DA. Colored boxes on the right indicate the relative concentration (from low to high) of corresponding proteins against each group under study.

Out of 12 bioactive isolates, we selected only six isolates based on the higher bioactivity (MIC and biofilm assays) (ASQP_29, ASQP_77 and ASQP_80), possible novel phylogenetic nature of a bioactive strain (ASQP_92, ASQP_98) and the absence or limited genome submissions in GenBank databases of a bioactive strain or its nearest possible phylogenetic ancestor (ASQP_78). Untargeted Label free quantitative proteomics was performed on these six selected bioactive *Streptomyces* isolates with the strain IDs ASQP_29, ASQP 77, ASQP_78, ASQP_80, ASQP_92 and ASQP_98. A total of 470 proteins were isolated from these bioactive isolates, run in triplicate. Using non-parametric one-way ANOVA with an adjusted *P* value cut-off of 0.05 and post-hoc tests, 122 proteins were identified as significant. Multivariate analytical approaches were applied to these significant proteins, yielding the following results. Unsupervised principal component analysis (PCA) generated 2D and 3D scores plots, showing a total segregation of ASQP_29 from the rest of the isolates. The five remaining isolates displayed mostly overlapping distribution of proteomic datapoints with some variability between the samples. The ellipse representing the confidence interval of 95% was largest in ASQP_92, indicating that this *Streptomyces* generated more variability in the proteome (Fig. 6A and Supplementary Fig. S3a).

Partial least square discriminant analysis (PLS-DA) was used to model differences more specifically between the isolates and to select specific features in the data (Fig. 6B and Supplementary Fig. S3b). 2D scores plots for PLS-DA showed more clear representation where ASQP_29 was clustered separately and distantly from other *Streptomyces* isolates. Greater variability was seen in ASQP_92 followed by ASQP_29, while comparatively less variability was seen in ASQP_80 and ASQP_98 (Fig. 6B). Hierarchical clustering analysis using a heatmap of the top 60 proteins (*P* < 0.05) showed clear segregation of ASQP_29 from ASQP_80 (Fig. 6C). Most of the proteins upregulated in ASQP_29 were downregulated in ASQP_80, and proteins downregulated in ASQP_29 were upregulated in ASQP_80. The expression profile of ASQP_77 was probably different from the other samples, as almost all the proteins under study were downregulated. The expression of ASQP_78, ASQP_92 and ASQP_98 were somewhat similar, with little differential expression profiling of few proteins. Furthermore, ASQP_78, ASQP_92 and ASQP_98 were more similar to ASQP_77 than to ASQP_29 and ASQP_80. A Variable Importance in Projection (VIP) plot was drawn to project important features identified among differentially expressed proteins (DEPs) by PLS-DA (Fig. 6D). The graph shows the relative contribution of proteins to the variance between different *Streptomyces* isolates. Proteins with high VIP scores, such as atpE, SCO1213 and STRIP9103_0627, indicated a greater contribution to the group separation. Many of these features involved in group separation between the selected isolates contribute to *Streptomyces* antibiotic response and stress tolerance.

**Figure 7.**
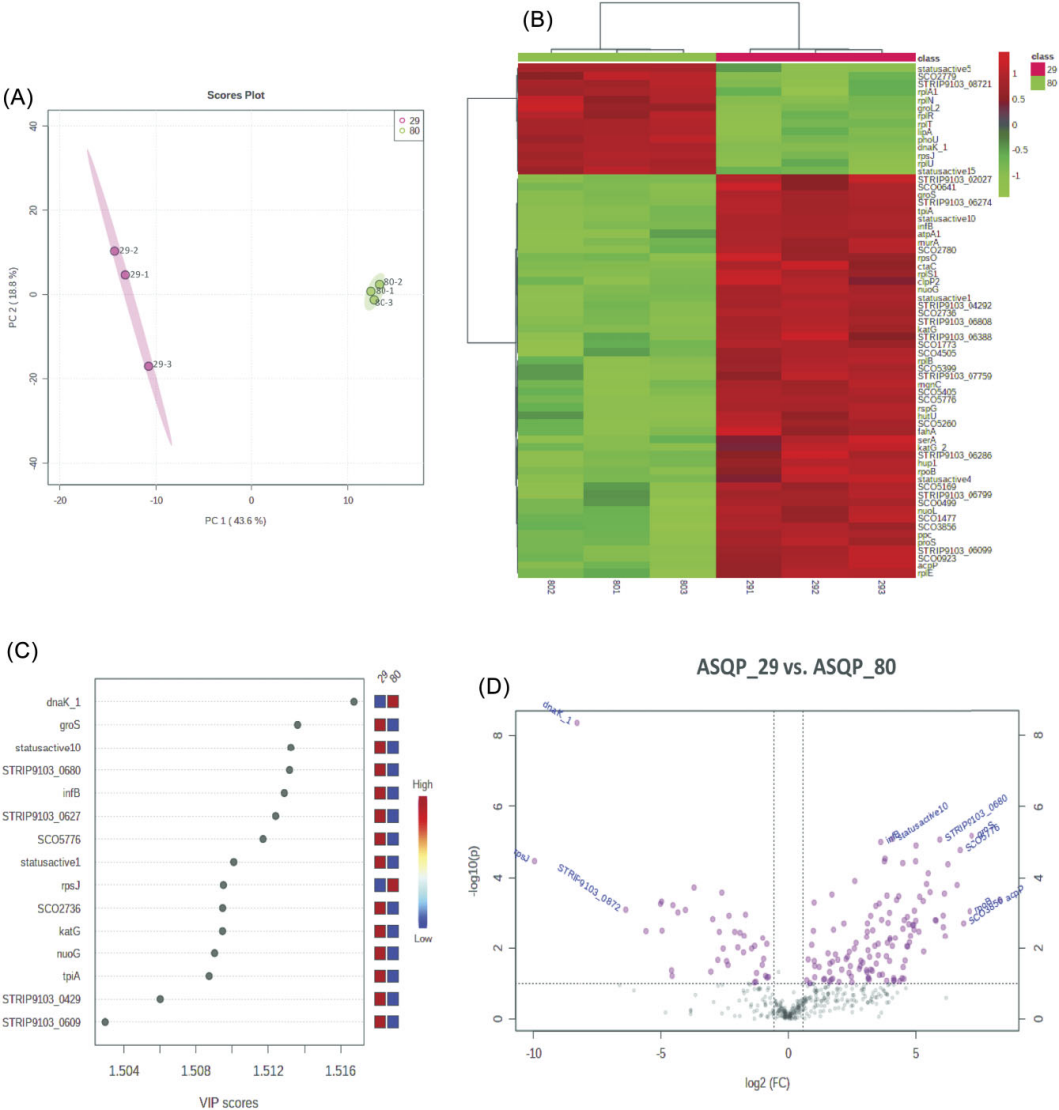
Proteomic phenotype of two highly bioactive *Streptomyces* (*Streptomyces* sp. ASQP_29 and *Streptomyces* sp. ASQP_80). (A) 2D scores plot for partial least square discriminant analysis (PLS-DA) using MetaboAnalyst 5.0. A five-component model was performed by PLS-DA. The plot shows two different samples in different colors. Ovals display 95% confidence intervals for both the samples. The diagram shows a scatterplot for two components of the greatest variation. Similar observations will fall close to each other and display a cluster-like pattern. Component 1 (x-axis) contains 43.6% of total variation and Component 2 contains 18.8%; 62.4% of total variance is observed by the first two components. (B) Hierarchical clustering heatmap of 65 *Streptomyces* proteins; hierarchical clustering was performed for two different *Streptomyces*, comparing 65 Benjamini–Hochberg FDR correction t-test *P* value (<0.05) passing proteins. For clustering, MetaboAnalyst 5.0 was used. The horizontal axis represents two samples analyzed in the study and the vertical axis is UniProt accessions for 65 proteins. On top of the heatmap are two different *Streptomyces* samples, each represented in a different color. A dendrogram for two different *Streptomyces*, each in triplicate, is shown on top of the heatmap and the protein dendrogram is on the left-hand side of the heatmap. Color gradient from dark green to dark red denotes lower to higher expression. (C) Variable Importance in Projection (VIP) plot displays the top 15 most important proteins identified by PLS-DA. Colored boxes on the right indicate the relative concentration (from low to high) of corresponding proteins against each group under study. (D) In this Volcano plot, the x-axis is the Log2 of linear fold change and the y-axis is the negative Log10 of the Benjamini–Hochberg-corrected t-test *P* value. The horizontal black dashed line designates a cut-off of 0.05 for the FDR-corrected *P* value. The plot displays the proteins that fall to the left and right of vertical black dashed lines and above the horizontal black dashed line (*P* < 0.05) as significant (proteins in colored dots), upregulated towards the right and downregulated towards the left in ASQP_29 versus ASQP_80. The top significant proteins are label+ed as shown in the plot.

We employed a systems biology approach to analyze the proteomes of two highly bioactive *Streptomyces* isolates with the strain IDs ASQP_29 (MIC 3.9 µg ml^–1^) and ASQP_80 (MIC 0.98 µg ml^–1^) (Fig. 7). A comparative expression analysis of the two isolates revealed 165 DEPs, with 125 upregulated and 40 downregulated in ASQP_29 compared with ASQP_80. PLS-DA analysis using a 2D plot showed a clear separation between the two samples, with more proteome variability in ASQP_29 (Fig. 7A). Heatmap clustering analysis of the expression profiles of the top 65 proteins showed contrasting expression levels between the two bioactive samples (Fig. 7B). Proteins from statusactive5 to statusactive15 were found to have increased expression in ASQP_80 but decreased expression in ASQP_29, while proteins listed after statusactive15 to the end (rpIE) were upregulated in ASQP_29 and downregulated in ASQP_80. This was consistent with the PCA and PLS-DA plots of all six samples, where ASQP_29 was clustered separately from ASQP_80 (Fig. 6C and Fig. 7B). Furthermore, feature identification using a VIP plot of the top 15 proteins, such as DnaK-1 (chaperone), groS (chaperonin), infB (Translation initiation factor IF-2) and rpsJ (30S ribosomal protein S10), revealed significant shifts in their expression potential and largely contributed to the group separation, with VIP features showing differential concentration gradients between the two samples (Fig. 7C). Volcano plot analysis (*P* < 0.05, FC > 1.5) showed that top significant proteins, such as DnaK-1 and rpsJ, among others, were downregulated, while proteins like groS and infB were upregulated in ASQP_29 compared with ASQP_80 (Fig. 7D). Finally, using the UniProt search engine, the gene ontology annotation terms of these proteins supported their involvement in biological processes related to response to cold and chaperone-mediated protein folding.

## Discussion

Our study on *Streptomyces* growth characteristics from high-altitude NWH showed that these microbes were maximally isolated using four different media types, that is, ISP2 (M3), SCA (Starch Casein Agar) (M6), CYPS (M7) and KMM (M8). Looking into the composition of these media types, soluble starch and dextrose can best serve as the primary source of glucose; yeast extract, malt extract and salt supplements in lower concentrations can serve as the source of amino acids, vitamins, minerals and other nutrients that support the microbial growth of diverse *Streptomyces* from these high-altitude regions. Thus, the optimum media composition of yeast and malt extracts in specific proportion with other ingredients such as peptones, limited salts and sugars (soluble starch and dextrose) can serve as the suitable culture medium for isolation of *Streptomyces* from high-altitude NWH regions. However, the specific concentrations of each component in the media, as well as any additional supplements or variations, might be adjusted based on the particular strains of *Streptomyces* or the environmental conditions of the high-altitude regions from which they are isolated. Experimental optimization and fine tuning may be necessary to achieve the best growth conditions for these bacteria.

Our *Streptomyces* species diversity assessment and application of inhibitory bioassays showed that the NWH microbiome is a promising source of bioactive *Streptomyces* and has the potential to isolate novel cultures. When compared, although the culturable biodiversity of these *Streptomyces* is less as expected, the potential for inhibition and isolation of novel taxa by these high-altitude ranges signifies the importance of exploration to yield further species diversity and novelty and tap the untapped biochemical potential to produce novel biochemical scaffolds. Low *Streptomyces* culturable species diversity from these Himalayan ranges may be attributed to the low temperatures, oligotrophy, dispersal limitation and glaciated soils of these sampling sites. The microclimate ice cold temperatures and historical patterns of glaciations in Himalaya and adjacent mountains might have limited the time for *Streptomyces* speciation, decreased the phylogenetic diversity and increased the phylogenetic clustering of this genus (Hewitt 1996). Phylogenetic clustering of NWH and species commonness among different sites may be attributed to their equitable ecological and climate conditions, with almost all the sites bearing a similar climate and vegetation patterns. Further, there is negative correlation between *Streptomyces* phylogenetic diversity and altitude/latitude (Andam et al. 2016). Also, varied *Streptomyces* culturable β-diversity and biochemical potential was observed among different sampling sites and may be because of the combination of dispersal limitation shaping *Streptomyces* diversity, bioactivity and selective environmental pressure particular to specific sampling sites (Eisenlord et al. 2012). The habitat filtering better known to microbiologists as the Baas Becking hypothesis—“Everything is everywhere, but, the environment selects”—applies well to *Streptomyces* from NWH habitats (van der Gast 2015). The high altitudes, cold temperatures and nutrient constraints limit the diversity and increase the selective and competitive pressures for the production of biochemically active scaffolds and novel bioactives. Further 16S rRNA lacks taxonomic resolution below the genus level and is an incomplete phylogenetic marker for proper *Streptomyces* identification. Use of a combination of phylogenetic markers (subunit B of DNA gyrase-gyrB, heat shock protein-hsp70 or DnaK, β subunit of bacterial RNA polymerase-rpoB, beta chain of tryptophan synthase-trpB and ATP-dependent DNA helicase-recG) along with universal 16S rRNA gene, or even more advanced although costly in silico-based phylogenomic approaches (Average Nucleotide Identity, DNA-DNA Hybridization), will help in proper species identification and pattern distribution of *Streptomyces* from NWH. Based on the dry cell mass quantification, media optimization of potential *Streptomyces* spp. displayed their varying growths and highlights the use of specific media for large-scale culture cultivation in the drug production process.

The proteomic analysis of two highly bioactive isolates of *Streptomyces* from high-altitude NWH, namely, ASQP_29 and ASQP_80, has shed light on their potential as a rich source of bioactive natural products. The use of a systems biology approach in the proteomic analysis revealed 165 DEPs between the two isolates, with more proteome variability observed in ASQP_29. These DEPs are involved in response to cold and chaperone-mediated protein folding, highlighting the unique adaptations of these *Streptomyces* to extreme environmental conditions. Furthermore, feature identification using a VIP plot of the top 15 proteins revealed significant shifts in their expression potential and largely contributed to the group separation with VIP features showing differential concentration gradients between the two samples. These results suggest that the two isolates have different protein expression profiles that may contribute to their respective bioactivity. The identification of specific proteins that are differentially expressed between the two isolates provides a valuable insight into the biological processes involved in their bioactivity. Further studies can focus on investigating the functional roles of these proteins and their potential applications in drug discovery and development.

In general, NWH reflects tremendous potential for bioactive exploration of *Streptomyces*. In our study we showed the diversity analysis, bioactivity and distribution pattern of culturable *Streptomyces* across different NWH regions. We also studied the proteomic expression profile of selective bioactive *Streptomyces* and the significance of some proteins in stress response and antibiotic regulation. The results encourage that further exploration of the NWH region with a larger sample size may provide more insights into *Streptomyces* diversification and proper pattern distribution. The exploration of such habitats may prove to be a valuable strategy in the search of more *Streptomyces* species for new natural molecules with antimicrobial properties.

## Supplementary Material

fiae026_Supplemental_Files

## Data Availability

All the sequencing data of 16S rDNA can be found at the NCBI's GenBank portal under accession numbers OQ290706-OQ290761.
