# Supplementary material for: Culturable Streptomyces spp. from high-altitude, oligotrophic North Western Himalaya: a comprehensive study on the diversity, bioactivity and insights into the proteome of potential species
Source: FEMS Microbiol Ecol. 2024 Mar 4;100(4):fiae026. doi: 10.1093/femsec/fiae026 (PMC10950047; doi:10.1093/femsec/fiae026)
Supplement: fiae026_Supplemental_Files [file fiae026_supplemental_files.zip › Supplementary_data Table_1 (2).docx]

| Sampling Site | Strains Isolated | Total Isolates | *Streptomyces* Isolates |
| --- | --- | --- | --- |
| S1 | ASQP 4, ASQP 9, ASQP 10, ASQP 12, ASQP 18, ASQP_29, ASQP 38, ASQP 40, ASQP 51^#^, ASQP 65, ASQP 71, ASQP 74^#^, ASQP 79, ASQP 87, ASQP 148, ASQP 213, ASQP 220 | 17 | 15 |
| S2 | ASQP 38a, ASQP 41, ASQP 46, ASQP 62, ASQP 67, ASQP 89, ASQP_92, ASQP 130, ASQP 145, ASQP 177, ASQP 192, ASQP a5^#^ | 12 | 11 |
| S3 | ASQP 5, ASQP 37, ASQP 52, ASQP 54, ASQP 75, ASQP 97, ASQP 128^#^, ASQP 135, ASQP 171, ASQP 209, ASQP 212 | 11 | 10 |
| S4 | ASQP 6, ASQP 13, ASQP 15, ASQP 19, ASQP 45, ASQP 57, ASQP 77, ASQP_78, ASQP_80, ASQP 94, ASQP_98, ASQP 123a, ASQP 142, ASQP a3^#^ | 14 | 13 |

**Supplementary Table 1.** Details of the strains isolated from different sampling sites. Non *Streptomyces* strains are marked with superscript #
