# Supplementary material for: Culturable Streptomyces spp. from high-altitude, oligotrophic North Western Himalaya: a comprehensive study on the diversity, bioactivity and insights into the proteome of potential species
Source: FEMS Microbiol Ecol. 2024 Mar 4;100(4):fiae026. doi: 10.1093/femsec/fiae026 (PMC10950047; doi:10.1093/femsec/fiae026)
Supplement: fiae026_Supplemental_Files [file fiae026_supplemental_files.zip › Supplementary_data Table_2 (1).docx]

**Supplementary Table 2.** Taxa identified from different sampling sites and their top blast hit/s with accession numbers and percentage identity level.

| **S. No.** | **Sequence_ID** | **Organism** | **Strain** | **Sampling site** | **Top Blast hit(s)** |
| --- | --- | --- | --- | --- | --- |
| 1 | ASQP_4 | *Streptomyces sp.* | ASQP 4 | S1 | Streptomyces badius\|NRRL B-2567\|AY999783\|100 |
|  |  |  |  |  | Streptomyces globisporus\|NBRC 12867\|AB184203\|100\| |
|  |  |  |  |  | Streptomyces sindenensis\|NBRC 3399\|AB184759\|100\| |
|  |  |  |  |  | Streptomyces parvus\|NBRC 3388\|AB184756\|100\| |
|  |  |  |  |  | Streptomyces pluricolorescens\|NBRC 12808\|AB184162\|100\| |
|  |  |  |  |  | Streptomyces rubiginosohelvolus\|NBRC 12912\|AB184240\|100\| |
| 2 | ASQP_5 | *Streptomyces pratensis* | ASQP 5 | S3 | Streptomyces pratensis\|ch24\|JQ806215\|100\| |
| 3 | ASQP_6 | *Streptomyces sp.* | ASQP 6 | S4 | Streptomyces pseudovenezuelae\|DSM 40212\|KQ948163\|98.64\| |
| 4 | ASQP_9 | *Streptomyces sp.* | ASQP 9 | S1 | Streptomyces pratensis\|ch24\|JQ806215\|99.1\| |
| 5 | ASQP_10 | *Streptomyces sp.* | ASQP 10 | S1 | Streptomyces cacaoi subsp. asoensis\|NRRL B-6592\|DQ026644\|99.77\| |
| 6 | ASQP_12 | *Streptomyces sp.* | ASQP 12 | S1 | Streptomyces badius\|NRRL B-2567\|AY999783\|99.09\| |
|  |  |  |  |  | Streptomyces puniceus\|NBRC 12811\|AB184163\|99.09\| |
|  |  |  |  |  | Streptomyces parvus\|NBRC 3388\|AB184756\|99.09\| |
|  |  |  |  |  | Streptomyces pluricolorescens\|NBRC 12808\|AB184162\|99.09\| |
|  |  |  |  |  | Streptomyces rubiginosohelvolus\|NBRC 12912\|AB184240\|99.09\| |
|  |  |  |  |  | Streptomyces globisporus\|NBRC 12867\|AB184203\|99.09\| |
|  |  |  |  |  | Streptomyces sindenensis\|NBRC 3399\|AB184759\|99.09\| |
| 7 | ASQP_13 | *Streptomyces sp.* | ASQP 13 | S4 | Streptomyces canus\|DSM 40017\|KQ948708\|99.68\| |
| 8 | ASQP_15 | *Streptomyces sp.* | ASQP 15 | S4 | Streptomyces badius\|NRRL B-2567\|AY999783\|99.03\| |
|  |  |  |  |  | Streptomyces parvus\|NBRC 3388\|AB184756\|99.03\| |
|  |  |  |  |  | Streptomyces sindenensis\|NBRC 3399\|AB184759\|99.03\| |
| 9  9 | ASQP_18  ASQP_18 | *Streptomyces sp.*  *Streptomyces sp.* | ASQP 18  ASQP 18 | S1  S1 | Streptomyces badius\|NRRL B-2567\|AY999783\|99.16\| |
|  |  |  |  |  | Streptomyces puniceus\|NBRC 12811\|AB184163\|99.16\| |
|  |  |  |  |  | Streptomyces parvus\|NBRC 3388\|AB184756\|99.16\| |
|  |  |  |  |  | Streptomyces pluricolorescens\|NBRC 12808\|AB184162\|99.16\| |
|  |  |  |  |  | Streptomyces rubiginosohelvolus\|NBRC 12912\|AB184240\|99.16\| |
|  |  |  |  |  | Streptomyces globisporus\|NBRC 12867\|AB184203\|99.16\| |
|  |  |  |  |  | Streptomyces sindenensis\|NBRC 3399\|AB184759\|99.16\| |
| 10 | ASQP_19 | *Streptomyces sp.* | ASQP 19 | S4 | Streptomyces novaecaesareae\|NRRL B-1267\|JNWQ01000181\|99.64\| |
| 11 | ASQP_29 | *Streptomyces sp.* | ASQP_29 | S1 | Streptomyces arenae\|ISP 5293\|AJ399485\|99.64\| |
|  |  |  |  |  | Streptomyces violarus\|NBRC 13104\|AB184316\|99.57\| |
| 12 | ASQP_37 | *Streptomyces exfoliatus* | ASQP 37 | S3 | Streptomyces exfoliatus\|NRRL B-2924\|JNZP01000081\|99.42\| |
| 13 | ASQP_38 | *Streptomyces sp.* | ASQP 38 | S1 | Streptomyces setonii\|NRRL ISP-5322\|MUNB01000146\|100\| |
|  |  |  |  |  | Streptomyces anulatus\|NRRL B-2000\|DQ026637\|100\| |
|  |  |  |  |  | Streptomyces pratensis\|ch24\|JQ806215\|100\| |
| 14 | ASQP_38a | *Streptomyces sp.* | ASQP 38a | S2 | Streptomyces microflavus\|NBRC 13062\|AB184284\|100\| |
|  |  |  |  |  | Streptomyces fulvorobeus\|NBRC 15897\|AB184711\|100\| |
| 15 | ASQP_40 | *Streptomyces sp.* | ASQP 40 | S1 | Streptomyces setonii\|NRRL ISP-5322\|MUNB01000146\|99.78\| |
| 16 | ASQP_41 | *Streptomyces rishiriensis* | ASQP 41 | S2 | Streptomyces rishiriensis\|NBRC 13407\|AB184383\|100\| |
| 17 | ASQP_45 | *Streptomyces rishiriensis* | ASQP 45 | S4 | Streptomyces rishiriensis\|NBRC 13407\|AB184383\|99.93\| |
| 18 | ASQP_46 | *Streptomyces rhizosphaerihabitans* | ASQP 46 | S2 | Streptomyces rhizosphaerihabitans\|JR-35\|HQ267983\|99.85\| |
| 19 | ASQP_48 | *Streptomyces rishiriensis* | ASQP 48 | L2 | Streptomyces rishiriensis\|NBRC 13407\|AB184383\|99.92\| |
| 20 | ASQP_51 | *Microbacterium algeriense* | ASQP 51 | S1 | Microbacterium algeriense\|G1\|MK480726\|99.93\| |
| 21 | ASQP_52 | *Streptomyces sp.* | ASQP 52 | S3 | Streptomyces microflavus\|NBRC 13062\|AB184284\|100\| |
|  |  |  |  |  | Streptomyces fulvorobeus\|NBRC 15897\|AB184711\|100\| |
| 22 | ASQP_54 | *Streptomyces sp.* | ASQP 54 | S3 | Streptomyces olivochromogenes\|DSM 40451\|KQ948511\|99.39\| |
| 23 | ASQP_57 | *Streptomyces sp.* | ASQP 57 | S4 | Streptomyces pratensis\|ch24\|JQ806215\|99.64\| |
| 24 | ASQP_62 | *Streptomyces sp.* | ASQP 62 | S2 | Streptomyces pratensis\|ch24\|JQ806215\|99.64\| |
| 25 | ASQP_65 | *Streptomyces malachitospinus* | ASQP 65 | S1 | Streptomyces malachitospinus\|NBRC 101004\|AB249954\|99.93\| |
| 26 | ASQP_67 | *Streptomyces yanii* | ASQP 67 | S2 | Streptomyces yanii\|NBRC 14669\|AB006159\|100\| |
| 27 | ASQP_71 | *Streptomyces sp.* | ASQP 71 | S1 | Streptomyces hundungensis\|MBRL 251\|JN560157\|99.47\| |
| 28 | ASQP_74 | *Kitasatospora sp.* | ASQP 74 | S1 | Kitasatospora kifunensis\|IFO 15206\|AB022874\|98.79\| |
| 29 | ASQP_75 | *Streptomyces rishiriensis* | ASQP 75 | S3 | Streptomyces rishiriensis\|NBRC 13407\|AB184383\|99.92\| |
| 30 | ASQP_76 | *Streptomyces rishiriensis* | ASQP 76 | L1 | Streptomyces rishiriensis\|NBRC 13407\|AB184383\|99.85\| |
| 31 | ASQP_77 | *Streptomyces olivochromogenes* | ASQP 77 | S4 | Streptomyces olivochromogenes\|DSM 40451\|KQ948511\|100\| |
| 32 | ASQP_78 | *Streptomyces rhizosphaerihabitans* | ASQP_78 | S4 | Streptomyces rhizosphaerihabitans\|JR-35\|HQ267983\|99.07\| |
| 33 | ASQP_79 | *Streptomyces arenae* | ASQP 79 | S1 | Streptomyces arenae\|ISP 5293\|AJ399485\|99.64\| |
| 34 | ASQP_80 | *Streptomyces sp.* | ASQP_80 | S4 | Streptomyces pseudovenezuelae\|DSM 40212\|KQ948163\|99.33\| |
| 35 | ASQP_87 | *Streptomyces rishiriensis* | ASQP 87 | S1 | Streptomyces rishiriensis\|NBRC 13407\|AB184383\|100\| |
| 36 | ASQP_89 | *Streptomyces mirabilis* | ASQP 89 | S2 | Streptomyces mirabilis\|NBRC 13450\|AB184412\|99.84\| |
| 37 | ASQP_92 | *Streptomyces sp.* | ASQP_92 | S2 | Streptomyces hundungensis\|MBRL 251\|JN560157\|99.27\| |
| 38 | ASQP_94 | *Streptomyces sp.* | ASQP 94 | S4 | Streptomyces fagopyri\|QMT-28\|MN044908\|98.96\| |
| 39 | ASQP_97 | *Streptomyces yanii* | ASQP 97 | S3 | Streptomyces yanii\|NBRC 14669\|AB006159\|99.92\| |
| 40 | ASQP_98 | *Streptomyces sp.* | ASQP_98 | S4 | Streptomyces mirabilis\|NBRC 13450\|AB184412\|99.77\| |
| 41 | ASQP_123a | *Streptomyces sp.* | ASQP 123a | S4 | Streptomyces pseudovenezuelae\|DSM 40212\|KQ948163\|98.71\| |
| 42 | ASQP_128 | *Microbacterium algeriense* | ASQP 128 | S3 | Microbacterium algeriense\|G1\|MK480726\|99.77\| |
| 43 | ASQP_130 | *Streptomyces sp.* | ASQP 130 | S2 | Streptomyces globisporus\|NBRC 12867\|AB184203\|100\| |
|  |  |  |  |  | Streptomyces pluricolorescens\|NBRC 12808\|AB184162\|100\| |
|  |  |  |  |  | Streptomyces rubiginosohelvolus\|NBRC 12912\|AB184240\|100\| |
| 44 | ASQP_135 | *Streptomyces sp.* | ASQP 135 | S3 | Streptomyces badius\|NRRL B-2567\|AY999783\|100\| |
|  |  |  |  |  | Streptomyces globisporus\|NBRC 12867\|AB184203\|100\| |
|  |  |  |  |  | Streptomyces sindenensis\|NBRC 3399\|AB184759\|100\| |
|  |  |  |  |  | Streptomyces parvus\|NBRC 3388\|AB184756\|100\| |
|  |  |  |  |  | Streptomyces pluricolorescens\|NBRC 12808\|AB184162\|100\| |
|  |  |  |  |  | Streptomyces rubiginosohelvolus\|NBRC 12912\|AB184240\|100\| |
| 45 | ASQP_142 | *Streptomyces sp.* | ASQP 142 | S4 | Streptomyces badius\|NRRL B-2567\|AY999783\|100\| |
|  |  |  |  |  | Streptomyces globisporus\|NBRC 12867\|AB184203\|100\| |
|  |  |  |  |  | Streptomyces sindenensis\|NBRC 3399\|AB184759\|100\| |
|  |  |  |  |  | Streptomyces parvus\|NBRC 3388\|AB184756\|100\| |
|  |  |  |  |  | Streptomyces pluricolorescens\|NBRC 12808\|AB184162\|100\| |
|  |  |  |  |  | Streptomyces rubiginosohelvolus\|NBRC 12912\|AB184240\|100\| |
| 46 | ASQP_145 | *Streptomyces microflavus* | ASQP 145 | S2 | Streptomyces microflavus\|NBRC 13062\|AB184284\|100\| |
|  |  |  |  |  | Streptomyces fulvorobeus\|NBRC 15897\|AB184711\|100\| |
| 47 | ASQP_148 | *Streptomyces sp.* | ASQP 148 | S1 | Streptomyces badius\|NRRL B-2567\|AY999783\|100\| |
|  |  |  |  |  | Streptomyces globisporus\|NBRC 12867\|AB184203\|100\| |
|  |  |  |  |  | Streptomyces sindenensis\|NBRC 3399\|AB184759\|100\| |
|  |  |  |  |  | Streptomyces parvus\|NBRC 3388\|AB184756\|100\| |
|  |  |  |  |  | Streptomyces pluricolorescens\|NBRC 12808\|AB184162\|100\| |
|  |  |  |  |  | Streptomyces rubiginosohelvolus\|NBRC 12912\|AB184240\|100\| |
| 48 | ASQP_171 | *Streptomyces sp.* | ASQP 171 | S3 | Streptomyces setonii\|NRRL ISP-5322\|MUNB01000146\|100\| |
|  |  |  |  |  | Streptomyces anulatus\|NRRL B-2000\|DQ026637\|100\| |
|  |  |  |  |  | Streptomyces pratensis\|ch24\|JQ806215\|100\| |
| 49 | ASQP_177 | *Streptomyces sanglieri* | ASQP 177 | S2 | Streptomyces sanglieri\|NBRC 100784\|AB249945\|99.93\| |
| 50 | ASQP_192 | *Streptomyces pulveraceus* | ASQP 192 | S2 | Streptomyces pulveraceus\|LMG 20322\|AJ781377\|100\| |
| 51 | ASQP_209 | *Streptomyces sp.* | ASQP 209 | S3 | Streptomyces badius\|NRRL B-2567\|AY999783\|100\| |
|  |  |  |  |  | Streptomyces globisporus\|NBRC 12867\|AB184203\|100\| |
|  |  |  |  |  | Streptomyces sindenensis\|NBRC 3399\|AB184759\|100\| |
|  |  |  |  |  | Streptomyces parvus\|NBRC 3388\|AB184756\|100\| |
|  |  |  |  |  | Streptomyces pluricolorescens\|NBRC 12808\|AB184162\|100\| |
|  |  |  |  |  | Streptomyces rubiginosohelvolus\|NBRC 12912\|AB184240\|100\| |
| 52 | ASQP_212 | *Streptomyces microflavus* | ASQP 212 | S3 | Streptomyces microflavus\|NBRC 13062\|AB184284\|100\| |
|  |  |  |  |  | Streptomyces fulvorobeus\|NBRC 15897\|AB184711\|100\| |
| 53 | ASQP_213 | *Streptomyces alboflavus* | ASQP 213 | S1 | Streptomyces alboflavus\|NRRL B-2373\|JNXT01000131\|100\| |
| 54 | ASQP_220 | *Streptomyces flavofungini* | ASQP 220 | S1 | Streptomyces flavofungini\|NBRC 13371\|AB184359\|100\| |
| 55 | ASQP_a3 | *Nocardiopsis sp.* | ASQP a3 | S4 | Nocardiopsis dassonvillei subsp. dassonvillei\|DSM 43111\|ABUI01000017\|98.55\| |
| 56 | ASQP_a5 | *Nocardiopsis dassonvillei subsp. Dassonvillei* | ASQP a5 | S2 | Nocardiopsis dassonvillei subsp. dassonvillei\|DSM 43111\|ABUI01000017\|99.93\| |
