# Supplementary material for: Culturable Streptomyces spp. from high-altitude, oligotrophic North Western Himalaya: a comprehensive study on the diversity, bioactivity and insights into the proteome of potential species
Source: FEMS Microbiol Ecol. 2024 Mar 4;100(4):fiae026. doi: 10.1093/femsec/fiae026 (PMC10950047; doi:10.1093/femsec/fiae026)
Supplement: fiae026_Supplemental_Files [file fiae026_supplemental_files.zip › Supplementary_data Table_3 (1).docx]

**Supplementary Table 3.** Taxonomic identification using 16S rDNA based analysis and details of Actinomycetota/Actinobacteria (majorly *Streptomyces*) isolated from different sites.

| S. No. | NCBI Seq. ID | Seq.  Length (bp) | Sampling Site | Organism | Strain | GenBank acc. no. |
| --- | --- | --- | --- | --- | --- | --- |
| 1 | ASQP_4 | 1425 | S1 | *Streptomyces sp.* | ASQP 4 | OQ290706 |
| 2 | ASQP_5 | 1365 | S3 | *Streptomyces pratensis* | ASQP 5 | OQ290707 |
| 3 | ASQP_6 | 1176 | S4 | *Streptomyces sp.* | ASQP 6 | OQ290708 |
| 4 | ASQP_9 | 1356 | S1 | *Strepromyces sp.* | ASQP 9 | OQ290709 |
| 5 | ASQP_10 | 879 | S1 | *Streptomyces sp.* | ASQP 10 | OQ290710 |
| 6 | ASQP_12 | 1321 | S1 | *Streptomyces sp.* | ASQP 12 | OQ290711 |
| 7 | ASQP_13 | 1254 | S4 | *Streptomyces sp.* | ASQP 13 | OQ290712 |
| 8 | ASQP_15 | 1344 | S4 | *Streptomyces sp.* | ASQP 15 | OQ290713 |
| 9 | ASQP_18 | 1306 | S1 | *Streptomyces sp.* | ASQP 18 | OQ290714 |
| 10 | ASQP_19 | 1390 | S4 | *Streptomyces sp.* | ASQP 19 | OQ290715 |
| 11 | ASQP_29 | 1353 | S1 | *Streptomyces sp.* | ASQP_29 | OQ290716 |
| 12 | ASQP_37 | 1389 | S3 | *Streptomyces exfoliatus* | ASQP 37 | OQ290717 |
| 13 | ASQP_38 | 1401 | S1 | *Streptomyces sp.* | ASQP 38 | OQ290718 |
| 14 | ASQP_38a | 1394 | S2 | *Streptomyces sp.* | ASQP 38a | OQ290719 |
| 15 | ASQP_40 | 1409 | S1 | *Streptomyces sp.* | ASQP 40 | OQ290720 |
| 16 | ASQP_41 | 1303 | S2 | *Streptomyces rishiriensis* | ASQP 41 | OQ290721 |
| 17 | ASQP_45 | 1348 | S4 | *Streptomyces rishiriensis* | ASQP 45 | OQ290722 |
| 18 | ASQP_46 | 1298 | S2 | *Streptomyces rhizosphaerihabitans* | ASQP 46 | OQ290723 |
| 19 | ASQP_48 | 1303 | L2 | *Streptomyces rishiriensis* | ASQP 48 | OQ290724 |
| 20 | ASQP_51 | 1355 | S1 | *Microbacterium algeriense* | ASQP 51 | OQ290725 |
| 21 | ASQP_52 | 1394 | S3 | *Streptomyces sp.* | ASQP 52 | OQ290726 |
| 22 | ASQP_54 | 1311 | S3 | *Streptomyces sp.* | ASQP 54 | OQ290727 |
| 23 | ASQP_57 | 1401 | S4 | *Streptomyces sp.* | ASQP 57 | OQ290728 |
| 24 | ASQP_62 | 1420 | S2 | *Streptomyces sp.* | ASQP 62 | OQ290729 |
| 25 | ASQP_65 | 1385 | S1 | *Streptomyces malachitospinus* | ASQP 65 | OQ290730 |
| 26 | ASQP_67 | 1314 | S2 | *Streptomyces yanii* | ASQP 67 | OQ290731 |
| 27 | ASQP_71 | 1333 | S1 | *Streptomyces sp.* | ASQP 71 | OQ290732 |
| 28 | ASQP_74 | 1329 | S1 | *Kitasatospora sp.* | ASQP 74 | OQ290733 |
| 29 | ASQP_75 | 1289 | S3 | *Streptomyces rishiriensis* | ASQP 75 | OQ290734 |
| 30 | ASQP_76 | 1322 | L1 | *Streptomyces rishiriensis* | ASQP 76 | OQ290735 |
| 31 | ASQP_77 | 1293 | S4 | *Streptomyces olivochromogenes* | ASQP 77 | OQ290736 |
| 32 | ASQP_78 | 1436 | S4 | *Streptomyces rhizosphaerihabitans* | ASQP_78 | OQ290737 |
| 33 | ASQP_79 | 1396 | S1 | *Streptomyces arenae* | ASQP 79 | OQ290738 |
| 34 | ASQP_80 | 1351 | S4 | *Streptomyces sp.* | ASQP_80 | OQ290739 |
| 35 | ASQP_87 | 1328 | S1 | *Streptomyces rishiriensis* | ASQP 87 | OQ290740 |
| 36 | ASQP_89 | 1275 | S2 | *Streptomyces mirabilis* | ASQP 89 | OQ290741 |
| 37 | ASQP_92 | 1371 | S2 | *Streptomyces sp.* | ASQP_92 | OQ290742 |
| 38 | ASQP_94 | 1376 | S4 | *Streptomyces sp.* | ASQP 94 | OQ290743 |
| 39 | ASQP_97 | 1304 | S3 | *Streptomyces yanii* | ASQP 97 | OQ290744 |
| 40 | ASQP_98 | 1294 | S4 | *Streptomyces sp.* | ASQP_98 | OQ290745 |
| 41 | ASQP_123a | 1317 | S4 | *Streptomyces sp.* | ASQP 123a | OQ290746 |
| 42 | ASQP_128 | 1310 | S3 | *Microbacterium algeriense* | ASQP 128 | OQ290747 |
| 43 | ASQP_130 | 1356 | S2 | *Streptomyces sp.* | ASQP 130 | OQ290750 |
| 44 | ASQP_135 | 1445 | S3 | *Streptomyces sp.* | ASQP 135 | OQ290751 |
| 45 | ASQP_142 | 1442 | S4 | *Streptomyces sp.* | ASQP 142 | OQ290752 |
| 46 | ASQP_145 | 1443 | S2 | *Streptomyces microflavus* | ASQP 145 | OQ290753 |
| 47 | ASQP_148 | 1437 | S1 | *Streptomyces sp.* | ASQP 148 | OQ290754 |
| 48 | ASQP_171 | 1445 | S3 | *Streptomyces sp.* | ASQP 171 | OQ290755 |
| 49 | ASQP_177 | 1442 | S2 | *Streptomyces sanglieri* | ASQP 177 | OQ290756 |
| 50 | ASQP_192 | 1444 | S2 | *Streptomyces pulveraceus* | ASQP 192 | OQ290757 |
| 51 | ASQP_209 | 1425 | S3 | *Streptomyces sp.* | ASQP 209 | OQ290758 |
| 52 | ASQP_212 | 1439 | S3 | *Streptomyces microflavus* | ASQP 212 | OQ290759 |
| 53 | ASQP_213 | 1434 | S1 | *Streptomyces alboflavus* | ASQP 213 | OQ290760 |
| 54 | ASQP_220 | 1437 | S1 | *Streptomyces flavofungini* | ASQP 220 | OQ290761 |
| 55 | ASQP_a3 | 1176 | S4 | *Nocardiopsis sp.* | ASQP a3 | OQ290748 |
| 56 | ASQP_a5 | 1335 | S2 | *Nocardiopsis dassonvillei subsp. Dassonvillei* | ASQP a5 | OQ290749 |
