# Supplementary figures and images for: Culturable Streptomyces spp. from high-altitude, oligotrophic North Western Himalaya: a comprehensive study on the diversity, bioactivity and insights into the proteome of potential species
Source: FEMS Microbiol Ecol. 2024 Mar 4;100(4):fiae026. doi: 10.1093/femsec/fiae026 (PMC10950047; doi:10.1093/femsec/fiae026)

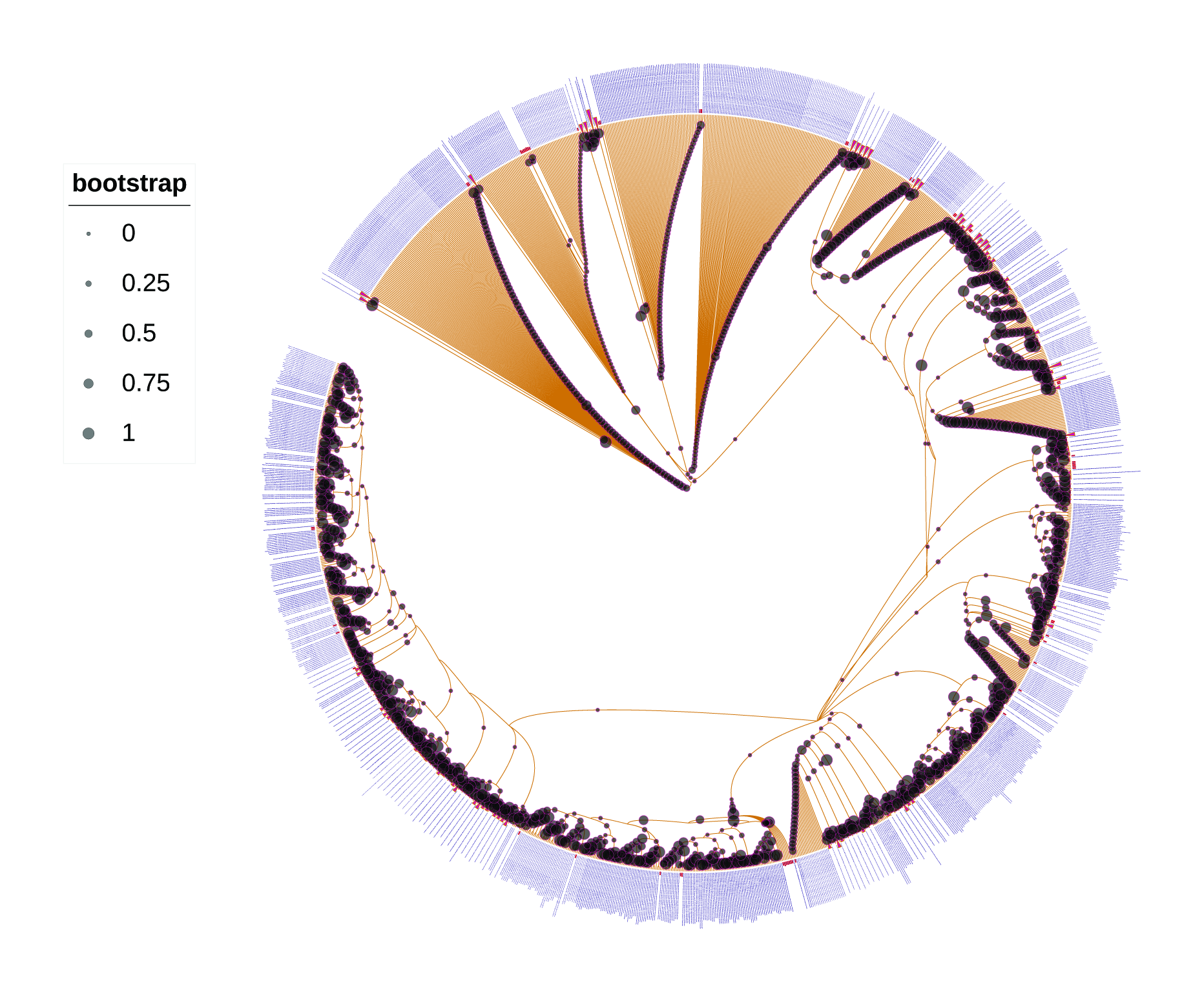

Supplement: fiae026_Supplemental_Files [file fiae026_supplemental_files.zip › required_Supplementary_data Fig._S1.tif]

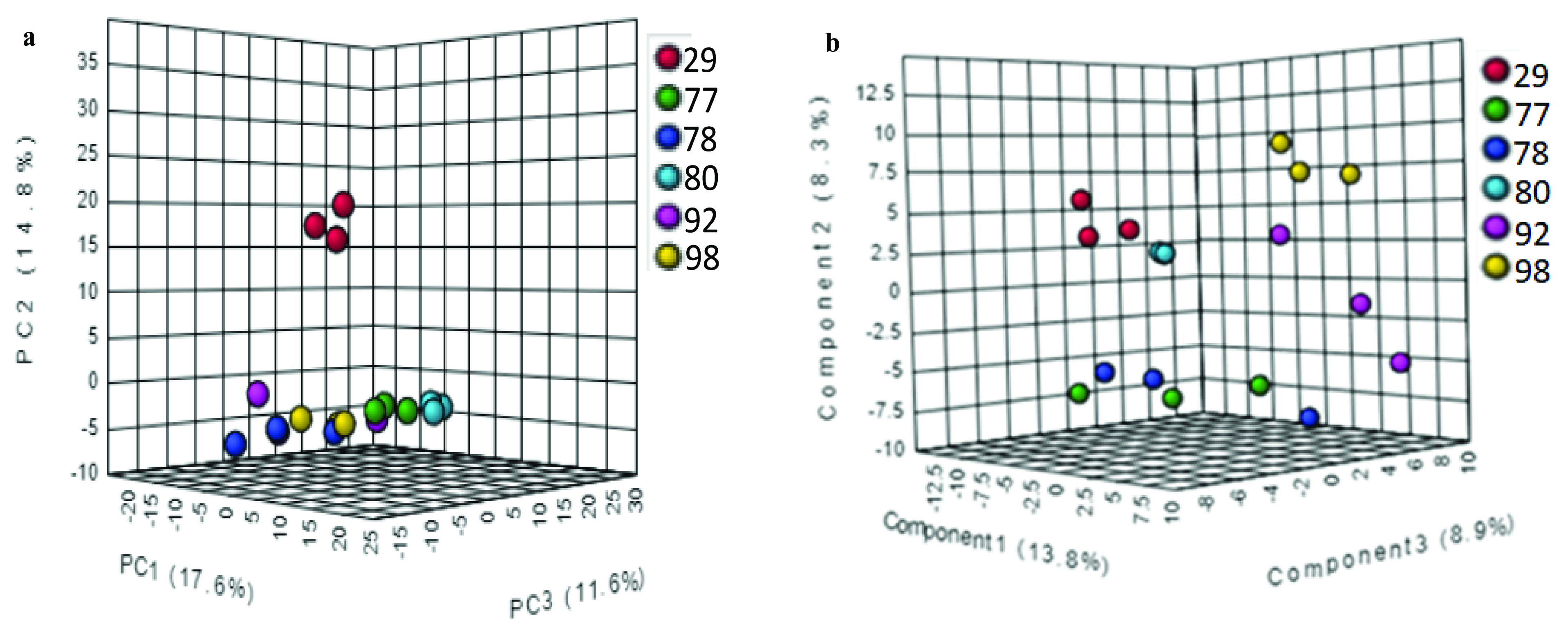

Supplement: fiae026_Supplemental_Files [file fiae026_supplemental_files.zip › Supplementary_data Fig._S3.tif]
